# Food Insecurity and Body Mass Index: A Longitudinal Mixed Methods Study, Chelsea, Massachusetts, 2009–2013

**DOI:** 10.5888/pcd12.150001

**Published:** 2015-08-06

**Authors:** Hong Chen Cheung, Aileen Shen, Sarah Oo, Hailu Tilahun, Marya J. Cohen, Seth A. Berkowitz

**Affiliations:** Author Affiliations: Aileen Shen, Injury Prevention at Boston Public Health Commission, Boston, Massachusetts; Sarah Oo, Community Health Department, Massachusetts General Hospital, Boston, Massachusetts; Hailu Tilahun, Department of Internal Medicine, Beth Israel Deaconess Hospital, Boston, Massachusetts; Marya J. Cohen, Adult Medicine, Massachusetts General Hospital, Chelsea Health Care Center, Chelsea, Massachusetts, Harvard Medical School, Boston, Massachusetts, Division of General Internal Medicine, Massachusetts General Hospital, Boston, Massachusetts; Seth A. Berkowitz, Harvard Medical School, Boston, Massachusetts, Division of General Internal Medicine, Massachusetts General Hospital, Boston, Massachusetts. Dr Chen is also affiliated with the Harvard Medical School, Boston, Massachusetts.

## Abstract

**Introduction:**

Cross-sectional studies show an association between food insecurity and higher body mass index (BMI), but this finding has not been evaluated longitudinally. Patient perspectives on food choice in resource-constrained environments are not well understood. The objective of this study was to evaluate the longitudinal association between food insecurity and BMI.

**Methods:**

This mixed methods study used both a retrospective matched cohort and focus groups. For the quantitative analysis, all patients in a community health center who reported food insecurity from October 2009 through March 2010 (n = 457) were followed through August 2013 and compared with controls matched by age, sex, and race/ethnicity (n = 1,974). We evaluated the association between food insecurity and change in BMI by using linear, mixed effects longitudinal models. The qualitative analysis included patients with food insecurity, stratified by BMI. Qualitative data were analyzed by using open coding and grounded theory.

**Results:**

The mean age of participants was 51 years; 61% were women, and 73% were Hispanic. Baseline BMI was similar in food insecure participants and matched controls. After adjustment in longitudinal analyses, food insecurity was associated with greater increase in BMI (0.15 kg/m^2^ per year more than controls, *P* < .001). Themes identified in 4 focus groups included attitudes and knowledge about food, food access, and food practices. Participants with BMI of 30 kg/m^2^ or less highlighted skills such as budgeting and portion control.

**Conclusion:**

Food insecurity is associated with increase in BMI. The skills of food insecure participants who were not obese, such as portion control and budgeting, may be useful in weight management interventions for vulnerable patients.

## Introduction

Obesity (body mass index [BMI] >30 kg/m^2^) affects more than one-third of American adults (1) and increases the risk for diabetes, heart disease, and all-cause mortality (2). The prevalence of obesity is higher among those with lower socioeconomic status (SES) (3). One possible mechanism for this is food insecurity, defined as a “lack of consistent access to nutritious foods in socially acceptable ways” (4). In 2013, approximately 15% of all American households were food insecure at some point during the year, and food insecurity was more common in households with lower incomes and members of racial/ethnic minority groups (5).

Food insecurity may paradoxically increase BMI by creating a “substitution effect” whereby inexpensive, energy-dense foods such as potato chips or processed meat replace healthier foods such as fresh produce and whole grains (6,7). Several cross-sectional studies have demonstrated an association between food insecurity, high BMI, and obesity (3,5,8–10). However, the relationship between food insecurity and change in BMI over time is unclear. Likewise, patient perspectives on food decisions in resource-constrained environments have not been well studied. Instances of positive deviance (11), that is, patients with food insecurity who nevertheless avoided obesity, may inform successful strategies to manage weight in vulnerable patients. The objective of this mixed methods study was to evaluate the longitudinal association between food insecurity and BMI by using a retrospective matched cohort design and focus groups.

## Methods

### Setting and study participants

This study was conducted at a community health center in Chelsea, Massachusetts. Chelsea is a diverse city where approximately 60% of the residents speak languages other than English and 62% of the population is Hispanic (12). Income below 200% of the federal poverty level was reported by 43% of health center patients (13).

From October 1, 2009, through March 31, 2010, all patients seen at the adult medicine practice were offered screening to assess food insecurity. Follow-up data on BMI were collected through August 31, 2013. All patients aged 18 years or older who visited the adult medicine clinic during the study period were eligible for the quantitative study. For the qualitative study, all patients with a measured BMI who reported food insecurity were eligible.

This study was approved by the Partners HealthCare Institutional Review Board, with waiver of informed consent for the secondary use of clinical data in the quantitative study. Focus group participants gave written informed consent.

### Assessment of food insecurity and BMI

Food insecurity was assessed at visit check-in (Appendix A) as part of determining eligibility for the Food for Family Program, which provides food pantry information, nutrition counseling, and other food resources to food insecure patients (14). A patient was considered to have reported food insecurity if he or she responded affirmatively to either of the 2 following questions: 1) In the past month, was there any day when you or anyone in your family went hungry because you did not have enough money for food? 2) Would you be interested in having someone contact you to talk more about getting food resources for you and your family?

The primary outcome for this study was BMI, taken by trained clinic staff at routine clinic visits. The BMI derived from the weight and height measurements taken during the visit when food insecurity was assessed was considered the baseline value.

For comparison in the quantitative analyses, we created a matched cohort from patients who visited the adult medicine practice during the time of the screening program but did not report food insecurity. This cohort was matched with food insecure patients on the basis of age, sex, and race/ethnicity in a 10:1 ratio. Controls could be matched to more than 1 food insecure participant.

### Covariates

We considered several covariates that may be associated with food insecurity, BMI, or both (3,8,10), which were abstracted from a repository of electronic health data. These included age, sex, race/ethnicity, educational attainment (<high school diploma vs ≥high school diploma), insurance (commercial, Medicare, Medicaid, or none/self-pay), and primary language spoken (English vs non-English). We used median household income, assessed at the block group level using United States Census data (15), to indicate neighborhood differences.

To evaluate the association between food insecurity and BMI, we compared participants who screened positive for food insecurity to their matched controls. Similar to data quality assurance procedures used in the National Health and Nutrition Examination Survey (NHANES) (16), values for weight or height that were above the national 99th percentile or below the 1st percentile were flagged for review. Of 40,013 observations, we excluded 7 weight values of less than 5 kg and 6 weight values greater than 640 kg as not physiologically reasonable. No height measurements were excluded. We then conducted descriptive statistics and compared the groups at baseline using χ^2^ tests for dichotomous variables and *t* tests or Wilcoxon tests (when distributions were non-normally distributed) for continuous variables. In this pragmatic study, we relied on data obtained in routine clinical care. This process resulted in an unbalanced design with varying intervals between measurements, so we used longitudinal linear mixed effect models to determine if changes in BMI (and weight) over time differed by food security status, using a time-by-food-security interaction term and accounting for repeated measures within patients with random effects modeling. We conducted both unadjusted longitudinal analyses and analyses adjusted for the covariates described above, including age, race/ethnicity, and sex to account for differences that persisted despite matching. All quantitative analyses were conducted with SAS version 9.3 (SAS Institute). Because weight has a curvilinear relationship with age, increasing through middle age and then decreasing among older adults (17), we modeled age with both a linear and a quadratic term.

During the study period, height was not consistently recorded in the electronic health record, which led to 24% of food insecure patients and 38% of matched controls lacking height data needed to calculate a BMI. To ensure that missing height data did not introduce bias, we conducted sensitivity analyses using weight in kilograms, which was available for all patients, as the outcome variable, in what were otherwise the same models used for the BMI analysis.

### Qualitative analysis

The purpose of the focus groups was to understand barriers to healthy eating among patients with food insecurity and to learn successful strategies to avoid obesity despite adverse circumstances (positive deviance). We developed a focus group guide by reviewing behavior theories from the health belief model (18), social cognitive theory (19), and the people and places framework (20). Some sample questions were adapted from the flexible consumer behavior survey module used in fielding NHANES 2009–2010 (21). The focus group guide was piloted and translated before use (Appendix B).

Patients who reported food insecurity and had BMI data available were selected at random and invited to participate the focus groups. Our maximum number of contact attempts was 3. Participants were stratified on the basis of BMI (BMI >30 kg/m^2^ vs ≤30 kg/m^2^) and primary language spoken (English vs Spanish). Prospective participants were offered lunch and $12 in grocery store coupons or $10 gift cards for participation.

We planned to have 4 focus groups, 1 for each stratum. However, because of limited participation, we completed 1 focus group among English-speaking participants with BMI >30 kg/m^2^ (n = 7), 2 English-language focus groups with participants with BMI ≤30 kg/m^2^ (n = 2 for each), and one Spanish-language focus group combining BMI strata (n = 10).

The focus groups were digitally recorded and then transcribed verbatim. From these records, emergent themes were identified by individual reviewers, who undertook open coding of the data. Next, coders met and reached consensus about themes. One source of influence for thinking about the themes as they emerged was the theory of people and places (20). This framework is an ecological model of health, which organizes factors that might support or thwart health. Key factors include attributes of people, including skills such as budgeting and portion control, and attributes of places, including local community organizations, or state and national policies and programs. Once themes emerged, we presented our findings to the Healthy Chelsea Coalition, a nonmedical community organization concerned with obesity in Chelsea, and community health care providers, who found these themes to be in accord with their experience.

## Results

### Quantitative results

Overall, 457 patients with food insecurity identified during the study period were matched to 1,974 patients in the comparison cohort. Compared with patients matched by age, sex, and race/ethnicity, food insecure patients were more likely to have not completed high school, have Medicaid insurance, and live in a census block group with lower income (median income of $33,272) (Table 1). Mean follow-up time was 3.2 years, and length of study follow-up was similar for the 2 groups; 105 food insecure patients (23.0%) and 363 control patients (18.4%) had a last recorded weight 1 year or more before the end of the study (χ^2^ *P* = .55 for difference between groups). At baseline, prevalence of obesity was high overall, but not significantly greater in food insecure, compared with control, patients (49.7% vs 45.2%, *P* = .14). Baseline BMI was also not greater in food insecure participants (30.9 kg/m^2^ vs 30.4 kg/m^2^, *P* = .11). Results for baseline weight (81.2 kg in food insecure vs 79.7 kg in matched controls, *P* = .14) were similar to those for BMI. By the end of the study, the prevalence of obesity was greater in participants who reported food insecurity at baseline (52.7% vs 44.7%, *P* = .001).

**Table 1:** Demographics Characteristics of Participants in a Study of Food Insecurity and Body Mass Index, Chelsea, Massachusetts, 2009–2013

Characteristic	Food Insecure (n = 457), % or Mean (SD)	Food Secure Matched Controls (n = 1,974), % or Mean (SD)	*P* Value^a^
**Age^b^, y**	50.6 (14.6)	51.9 (15.2)	.12
**Female^b^**	61.3	61.6	.91
**Race/ethnicity^b^**			
Non-Hispanic white	18.2	18.2	.99
Non-Hispanic black	5.3	5.5	
Hispanic	73.3	72.8	
Asian/other	3.3	3.5	
**Insurance**			
Commercial	41.4	52.1	.001
Medicare	22.5	18.4	
Medicaid	31.7	25.1	
None/self-pay	4.4	4.4	
**<High school diploma**	50.4	44.9	.04
**Census block group median household income, $**	33,272 (12,218)	35,287 (13,977)	.004
**English as primary language**	33.3	36.8	.16
**BMI^c^ >30 kg/m^2^**	49.7	45.2	.14
**Follow-up time, y**	3.2 (0.92)	3.2 (0.96)	.70

Abbreviations: BMI, body mass index; SD, standard deviation.

a All *P* values are from χ^2^ tests except age, median household income, and follow-up time, which are from Wilcoxon tests.

b Cohort matched on these variables.

c N = 340 for food insecure and 1,243 for food secure.

In unadjusted linear mixed models, patients who reported food insecurity had significantly greater gain in BMI over time than matched controls (increase in BMI 0.15 kg/m^2^ per year greater in food insecure patients, *P* < .001) (Table 2). When evaluating change in weight, results were similar to those for BMI (0.33 kg/y greater increase in patients reporting food insecurity, *P* < .001). After adjusting for age, sex, race/ethnicity, education level, health insurance, primary language spoken, and block group median household income, food insecurity remained significantly associated with greater increase in BMI (0.15 kg/m^2^ per year greater increase in food insecure patients, *P* <.001) and weight (0.31 kg/y greater increase, *P* < .001) (Table 2). Both the unadjusted and adjusted models predict that, although the BMI of control patients will decrease slightly over time, the BMI of food insecure patients will increase (Table 2).

**Table 2:** Unadjusted and Adjusted^a^ Longitudinal Results for Change in Body Mass Index (BMI) and Weight Over Time, by Food Security Status, Chelsea, Massachusetts, 2009–2013

Variable	Unadjusted BMI, kg/m^2^		Unadjusted Weight, kg		Adjusted^a^ BMI, kg/m^2^		Adjusted^a^ Weight, kg	
	Estimate (95% CI)	*P* Value	Estimate (95% CI)	*P* Value	Estimate (95% CI)	*P* Value	Estimate (95% CI)	*P* Value
Difference at baseline for food	0.39 (−0.35 to 1.14)	.30	1.17 (−0.69 to 3.03)	.22	0.20 (−0.61 to 1.01)	.63	0.69 (−1.18 to 2.55)	.47
Food secure	1 [Reference]							
Change per year^b^ (overall)	−0.12 (−0.14 to −0.09)	<.001	20.0 (−0.25 to −0.14	<.001	−0.13 (−0.16 to −0.10)	<.001	−0.22 (−0.28 to −0.16)	<.001
Differential change per year among food insecure participants^b^	0.15 (0.10 to 0.20)	<.001	0.33 (0.22 to 0.44)	<.001	0.15 (0.10 to 0.20)	<.001	0.31 (0.19 to 0.43)	<.001
Differential change among food secure controls	1 [Reference]							
Age (years)	—	—	—	—	0.47 (0.32 to 0.62)	<.001	1.21 (0.88 to 1.55)	<.001
Age^a^ × age (years^2^)	—	—	—	—	−0.0004 (−0.006 to −0.003)	<.001	−0.01 (−0.02 to −0.01)	<.001
**Sex**								
Female	—	—	—	—	0.36 (−0.36 to 1.07)	.33	−11.3 (−12.8 to −9.7)	<.001
Male	1 [Reference]							
**Race/ethnicity**								
Non-Hispanic black	—	—	—	—	0.29 (−1.37 to 1.85)	.77	2.65 (−1.01 to 6.31)	.16
Hispanic	—	—	—	—	−0.22 (−1.36 to 0.91)	.70	−4.15 (−6.66 to −1.65)	.001
Asian/other	—	—	—	—	−0.90 (−2.99 to 1.18)	.39	−7.65 (−12.22 to −3.08)	.001
Non-Hispanic white	1 [Reference]							
**Insurance**								
Medicare	—	—	—	—	0.87 (−0.20 to 1.95)	.11	2.74 (0.32 to 5.16)	.03
Medicaid	—	—	—	—	0.45 (−0.37 to 1.27)	.28	1.58 (−0.22 to 3.39)	.09
None/Self-pay	—	—	—	—	−0.14 (−1.96 to 1.68)	.88	0.05 (−3.69 to 3.79)	.98
Commercial	1 [Reference]							
**Education level**								
<High school diploma	—	—	—	—	0.74 (−0.02 to 1.50)	.06	0.04 (−1.61 to 1.69)	—
High school diploma	1 [Reference]							
**Census block group median household income (dollars)**	—	—	—	—	−0.00002 (−0.00004 to 0.000009)	.20	−0.00001 (−0.00007 to 0.00004)	.61
**English as primary language**	—	—	—	—	−0.72 (−1.66 to 0.23)	.14	−3.82 (−5.87 to −1.77)	<.001

Abbreviations: —, matching variable; CI, confidence interval.

a Adjusted for all variables in table.

b Because of the presence of a differential change by food security status (interaction) term in the regression models, to determine the change in BMI or weight per year for food insecure participants, the overall change in BMI (or weight) term was added to the differential change term. For food secure participants, only the overall term was used (because food secure participants are the reference group, their interaction term coefficient is 0). Thus, the models estimate that BMI and weight will decrease for food secure participants over time but will increase or stay the same for food insecure participants.

### Qualitative results

Participants, regardless of BMI, endorsed the importance of eating produce and avoiding highly processed and junk foods for maintaining health (Table 3, theme 1 quotes). For example, one participant stated, “I would like to buy more vegetables,” and another stated, “I would like to change the type of oil to olive or vegetarian.”

**Table 3:** Thematic Analysis of Qualitative Data From Focus Groups on Food Insecurity, Chelsea, Massachusetts, 2009–2013

Theme	Related Quotes
Attitude and knowledge about food	• I would like to buy more vegetables. (body mass index [BMI] >30 kg/m^2^ group) • My husband is Spanish and we have to have a decent meal at night, he deserves at least a good meal at night. (BMI >30 kg/m^2^ group) • Seasonal food is beneficial to taste. (BMI ≤30 kg/m^2^ group)
Economic issues	• It’s cheaper to buy in Wendy’s . . . the salad. (BMI >30 kg/m^2^ group) • Middle class disappeared. . . . I used to have a good earning job. (BMI >30 kg/m^2^ group) • [I] find social security a blessing. (BMI >30 kg/m^2^ group) • Food stamp is very little. . . . I don’t think I can get anything else (than food). (BMI ≤30 kg/m^2^ group) • I will eat before paying bills. (BMI >30 kg/m^2^ group) • Finances are the main barrier to eat healthy. (BMI ≤30 kg/m^2^ group) • Junk food is cheaper. (BMI ≤30 kg/m^2^ group) • [I am] dissatisfied with social security: when it comes out . . . how often a check is cut. (BMI ≤30 kg/m^2^ group)
Food access	• Pantries give unhealthy foods. (BMI >30 kg/m^2^ group) • Those without personal transportation go to closest supermarket even if it’s very crowded and the lines are very long, to save money. (BMI >30 kg/m^2^ group) • Farmers market during other time of year is great. Much cheaper, fresher, and you get variety. (BMI ≤30 kg/m^2^ group) • Beginning of the month eat better than end of the month. (BMI ≤30 kg/m^2^ group) • Insufficient access to/from supermarket. . . . Could benefit from shuttle services. (BMI ≤30 kg/m^2^ group)
Food practices	• Hard to plan out one month’s food. (BMI >30 kg/m^2^ group) • [I] buy meat in bulk and freeze it to save money. (BMI >30 kg/m^2^ group) • [I have] no time for cooking. (BMI >30 kg/m^2^ group) • [I] dilute milk with water to extend it. (BMI >30 kg/m^2^ group) • [I am] aware that can go overboard with food like eating too many cookies. (BMI ≤30 kg/m^2^ group) • My daughter-in-law knows how to use herbs for spices. . . . You need to match herbs with different food. (BMI ≤30 kg/m^2^ group) • I calculate the portion to cook and extend to next day. (BMI ≤30 kg/m^2^ group) • Sometimes stuff is pricey and we have to keep a budget. (BMI ≤30 kg/m^2^ group)

Economic issues influenced food access and food practices. Participants across all strata identified the expense of healthier foods, compared with less healthy choices (Table 3, theme 2 quotes). Others identified insufficient assistance programs, stating “Social security has not increased the check while the cost is higher,” and “Food stamps are not enough.”

The theme of insufficient resources carried over into difficulties with food access (Table 3, theme 3 quotes). Transportation was a struggle, and increases in commodity prices had downstream effects on food access. “[I]ncrease in gas prices [lead to] increase in produce prices, increase cost of transportation (car or cab),” and participants expressed frustration at the mismatch between receipt of assistance and access to opportunities to improve eating. For example, one stated, “[I] wish double and triple coupons came out at a more opportune time . . . around the time of the social security check”, and “pantries give unhealthy foods.” Additionally, participants reported that available assistance did not allow them to access healthier foods and instead often pushed them toward food they would like to avoid.

Food practices and choices were different for obese and nonobese participants (Table 3, theme 4 quotes). Participants without obesity identified discreet skills, such as budgeting, portion control, and cooking techniques, to cope with high prices and eat more healthily. Many quotes directly addressed their own practices as key to maintaining a healthy weight: “I calculate the portion to cook and extend to [the] next day.” By contrast, participants with obesity identified feeling unable to budget sufficiently and often resorted to convenience meals they knew to be unhealthy because they perceived no other options to cope with time and resource constraints. They identified the need for local or state assistance.

## Discussion

In this longitudinal mixed methods study, we found that, among a cohort matched by age, sex, and race/ethnicity with similar baseline BMI, baseline food insecurity was associated with greater increase in BMI during a mean follow-up time of 3.2 years. In fact, our models estimated that the BMI for food secure participants would decrease as it increased for food insecure participants. Exploring possible reasons for this difference among food insecure patients, participants endorsed wanting to eat healthy foods, and knowledge of healthy eating practices was high. However, participants highlighted economic barriers to healthy eating and the inadequacy of assistance programs, both with regard to the amount of assistance and the kinds of food available. Using a positive deviance approach by soliciting input from participants who succeeded in avoiding obesity despite adverse circumstances, we found that economic barriers may be overcome by skills such as portion control, budgeting, and cooking techniques.

The findings of this study are consistent with and expand those of prior work. Cross sectional studies have demonstrated an association between food insecurity and greater BMI (3,8–10). However, these studies could not evaluate time ordering between food insecurity and BMI. The finding that food insecure participants have greater increases in BMI in a cohort with similar baseline BMI and demographic characteristics is important for understanding the risk associated with food insecurity. Consistent with the theory of people and places (20), focus group participants looked beyond individual-level interventions as a solution to maintaining health. This included both social networks and the organizations and institutions of their community. The latter underscores the importance of community-based lifestyle interventions for healthy weight (22). Additionally, the feelings of being overwhelmed or unable to implement healthier eating practices despite knowledge of these strategies is consistent with low self-efficacy (23), which may be related to the detrimental cognitive impact of resource and time scarcity (24).

This study has several important implications for future weight management interventions in vulnerable patients, although these results should be confirmed in larger and more generalizable studies. Food insecurity screening has the potential to identify individuals at risk of BMI increase, and intervention programs can reduce food insecurity (25). However, because focus group participants identified some food assistance programs as sources of food that may lead to weight gain, BMI increase could also be an unintended consequence of some programs. In this study, knowledge of healthier eating strategies, such as increasing produce consumption and minimizing fast-food consumption was high among all participants. Participants without obesity identified discrete, teachable household management skills, such as portion control and budgeting, as protective factors, so interventions that pair food insecurity screening with skill-building interventions may be a promising strategy. However, given the additional socioeconomic barriers to maintaining health that food insecure patients face, these will need to be adapted to specific contexts. Strategies that use peer educators (26) or community health workers (27) may be particularly effective in this setting. Additionally, the focus group participants’ responses give impetus to proposed policy solutions, such as increasing the purchasing power of nutritional assistance benefits when spent on produce (28) or increasing the frequency of benefit distribution to combat end-of-month effects (29). Given the findings from this and other studies regarding patients maintaining health despite adverse circumstances, the community health center in this study is establishing a peer-mentoring program to reduce cardiovascular risk and working with the community benefit program in our health system and the Healthy Chelsea community organization to address neighborhood factors related to obesity. In addition, to promote healthy eating skills such as portion control, we have established a program, staffed by trained community volunteers and coordinated by the medical team, where recipes posted to a social media site by patients receive feedback for portion size and composition, from the perspective of maintaining a healthy weight.

The findings of this study should be interpreted in the context of several limitations. First, although the longitudinal design of this study overcomes limitations of prior cross-sectional work in evaluating the temporal relationship between food insecurity and BMI, we still made use of observational data and cannot establish a causal relationship. In particular, the possibility of reverse causation by enrollment in food programs with suboptimal food resources should be considered. Next, food insecurity is a household-level concept, but we have focused on individual patient data. However, prior work on food insecurity supports analyzing the data in this way (30). Additionally, we did not have access to some covariates that may have influenced food insecurity risk, including family size, employment, and income.

Records of negative screens for the program were not kept. Because of this, for the comparison group, we cannot know which patients reported food security, which patients declined screening, or the characteristics of each type of patient. However, because the group was drawn from the entire population of patients seen in the practice during the study period (including both those who declined screening and who reported no food insecurity), it represents an unbiased sample from the practice. This does mean, though, that the comparison group included a mix of patients who were truly food secure and some whose status was uncertain. Furthermore, because disadvantaged households cycle in and out of food insecurity over time (5), patients who were not experiencing food insecurity at baseline may have experienced it during the follow-up period. Both of these factors would diminish the observed association between food insecurity and BMI, so our results may be an underestimate. Next, height data needed to calculate BMI were missing for a significant number of patients. However, sensitivity analyses using weight as an outcome yielded similar results to analyses using BMI, suggesting that the missing data did not introduce significant bias. Additionally, this study was conducted in a single community, so results may not be generalizable to settings with different demographic compositions or social circumstances. However, this study does contribute data to understudied groups, such as patients reporting Hispanic ethnicity and limited English proficiency. Finally, lower-than-intended enrollment in focus groups may have limited our ability to reach content saturation, which may further affect generalizability.

These limitations are balanced by several strengths. The mixed methods design allowed us to understand the experiences of a subset of the same patients we observed longitudinally. The possible mechanisms identified in the focus groups as being related to the observed weight outcomes, such as food access and food practices, thus have a closer connection than can be achieved by studying 2 separate patient cohorts. Additionally, the use of a matched cohort drawn from the same disadvantaged area as the food insecure participants provided a comparison group that was similar to food insecure participants in ways beyond the matching factors. Finally, this study used a brief, pragmatic food insecurity screening instrument that also asked about interest in a nutritional assistance program. This is less precise than a longer epidemiological surveillance tool but does reduce respondent burden, more closely mirrors the conditions of routine practice, and identifies patients interested in intervention.

Food insecurity is an independent risk factor for rising BMI. Despite this, the experiences of food insecure patients who nevertheless avoid obesity point the way to skill-building and policy interventions that may modify this risk. Developing, evaluating, and implementing these interventions will be a key next step to reduce cardiovascular disease risk for socioeconomically vulnerable patients.

## Appendix A Food Security Screening Instrument

This file is available for download as a Microsoft Word document.

## Appendix B Focus Group Guide

This file is available for download as a Microsoft Word document.
