# Supplementary material for: Food Insecurity and Body Mass Index: A Longitudinal Mixed Methods Study, Chelsea, Massachusetts, 2009–2013
Source: Prev Chronic Dis. 2015 Aug 6;12:E125. doi: 10.5888/pcd12.150001 (PMC4565511; doi:10.5888/pcd12.150001)
Supplement: Supplementary file 1 [file 15_0001_AppendixA.doc]

**Appendix A. Food Security Screening Instrument**

1. In the past month, was there any day when you or anyone in your family went hungry because you did not have enough money for food?
2. We would like to know if you would be interested in talking with a counselor about healthy eating on a budget as well as finding out if you are eligible to receive additional food resources. The counselor will ask you some questions to see if you qualify for food resources like: food stamps or other free services like food pantries or hot meals. Would you be interested in having someone contact you to talk more about getting food resources for you and your family?
