# Supplementary material for: Food Insecurity and Body Mass Index: A Longitudinal Mixed Methods Study, Chelsea, Massachusetts, 2009–2013
Source: Prev Chronic Dis. 2015 Aug 6;12:E125. doi: 10.5888/pcd12.150001 (PMC4565511; doi:10.5888/pcd12.150001)
Supplement: Supplementary file 2 [file 15_0001_AppendixB.docx]

**Appendix B. Focus Group Guide**

Thank you for investing some time to come to share with us about food and health. As you know food is a basic need for human being.

FOOD ACCESS

1. What are the challenges you face to get food to feed your family?

Que problemas tiene para poder conseguir alimentos/comida para su familiar? Que impide que consigues

1. What type of resources help you get food?

De que manera consigue sus alimentos? Que recursos tienen?

1. Where do you get your food? (How far do you travel to get your food, is that the closest and if not why?)

Donde consigue su comida? (que tan lejos tiene que llegar para conseguir su comida, es ese el lugar mas cercano, y si no por que?)

PERCEPTIONS OF HEALTHY DIET

1. What types of foods do you like to have available for you and your family?  If you have kids in your family, what do you like your kids to eat?

Que tipo de alimentos te gusta tener disponible para usted y su familia? Si tiene niños, que le gustaria que coman?

1. Do you ever worry that you family is not eating healthy food? Why or why not?

Alguna vez se ha preocupado que su familia no esta comiendo saludable, por que si y por que no?

1. What type of meals do you think are healthy and good for you? Can you give examples?

Que tipo de comida usted piensa que son saludables y buenos para usted? Puede dar ejemplos?

FOOD CHOICES

1. How do you like your food be made?

Como le gusta su comida preparada?

1. If you had more money, would you change the type of food that your family eats? How so?

Si usted tiene suficiente dinero, usted cambiaria el tipo de comida que usted o su familia come? Como lo cambiaria?
